# Associations of working from home with job satisfaction, work-life balance, and working-model preferences

**DOI:** 10.3389/fpsyg.2023.1258750

**Published:** 2023-11-22

**Authors:** Tin Orešković, Milan Milošević, Bruna Kostelac Košir, Darko Horvat, Tomislav Glavaš, Antonio Sadarić, Carin-Isabel Knoop, Stjepan Orešković

**Affiliations:** ^1^Balliol College, University of Oxford, Oxford, United Kingdom; ^2^Andrija Štampar School of Public Health, University of Zagreb School of Medicine, Zagreb, Croatia; ^3^M+ Group Croatia Department of Human Resources, Zagreb, Croatia; ^4^IEDC Bled School of Management, Bled, Slovenia; ^5^Harvard Business School, Boston, MA, United States

**Keywords:** work from home, job satisfaction, work-life balance, COVID-19, gender

## Abstract

**Introduction:**

The COVID-19 pandemic forced many businesses to shift towards remote and hybrid working models. This study explored the association of the work-from-home model with employee satisfaction, work-life balance, and work-model preferences within MPlus Group, a leader in telework within the business process and technology outsourcing (BPTO) industry.

**Methods:**

We analyzed survey responses of 4,554 employees of MPlus Group across seven countries to assess the associations of working from home with job satisfaction, work-life balance, and preference regarding continuing to work from home.

**Results:**

Employees working within all models, and both women and men, reported high levels of job satisfaction and work-life balance, and most employees working from home expressed a desire to continue doing so.

**Discussion:**

Our findings suggest working from home does not lead to lower job satisfaction or work-life balance in the BPTO and similar industries. The study provides insights for organizations and policymakers navigating post-pandemic work dynamics. However, further research is needed to examine the long-term implications of remote work across diverse industries.

## 1 Introduction

In a period of transformation in work dynamics, organizations are increasingly concerned with the viability and sustainability of remote work arrangements. While remote work models have been around for many years, they have recently gained prominence as a method of organizing the workforce, especially due to the far-reaching impact of the COVID-19 pandemic. The prevalence of remote work has captured global headlines in addition to the business press, reporting on cases of significant migration resulting from remote work opportunities (Badger et al., 2023). Limited available academic research in this nascent field presents a broad range of findings.

While there is a lack of consensus regarding the appropriate term, ‘remote work,’ ‘telework,’ and ‘work from home,’ researchers have been exploring working outside the regular office space for decades before the COVID-19 pandemic. Since IBM sent five employees home and provided them with gigantic terminals, ‘telework’ has expanded to refer to a broader range of work locations, including the home, satellite offices, and other remote settings (e.g., Di Martino and Wirth, 1990). Similarly, ‘remote work’ as a term encompasses various work settings, such as working from home, co-working spaces, or other remote locations (e.g., Olson, 1983).

However, work from home implies that the employee’s primary workspace is within their own home, utilizing technology and digital tools to connect with colleagues and perform tasks remotely (e.g., Shamir and Salomon, 1985), potentially creating additional challenges for employee motivation and effectiveness.

Working from home some (here: hybrid) or all the time (here: simply working from home) can provide benefits such as enhanced autonomy, flexibility, and reduced commuting time, thereby improving job satisfaction, work-life balance as well as productivity, and reducing attrition. These positive effects were found in a study of engineers, marketing, and finance employees of a technology firm volunteering to enter a hybrid model randomized controlled trial (Bloom et al., 2022) and of customer service agents volunteering to enter a working-from-home randomized trial (Bloom et al., 2015). In contrast, some observational studies have found working from home to be associated with lower satisfaction and increased stress (Xiao et al., 2021; Lange and Kayser, 2022; Makridis and Schloetzer, 2022; Niebuhr et al., 2022). Bollestad et al. (2022) reported a reduction in employee exposure to bullying but also a rise in perceived loneliness, which was negatively associated with work engagement (Bollestad et al., 2022). Furthermore, Stanton and Tiwari (2021) considered the effect of remote workers’ need to occupy more space at home on housing consumption, thus cutting into savings and expanding the housing footprint. Higher utility bills are also a factor to consider.

An area of particular significance in equity and long-term human capital development are the potential implications of remote work for individuals with various family roles and responsibilities. Several studies accentuated adverse labor outcomes for women due to the requirement to work from home amid the COVID-19 pandemic, including reduced hours and regression of gender roles towards those of traditional models (Singh et al., 2022). Another study found that mothers of little children working from home spent 49 more minutes per day on housework than fathers with the same working model (Lyttelton et al., 2020). Kumaresan et al. (2022) assessed self-reported burnout among IT professionals working from home, finding that women, on average, reported higher burnout rates than men (Kumaresan et al., 2022).

Like employees, organizations have had to weigh the benefits and costs of the work-from-home model. Having employees at home can cut office space and resources expenses, which for some organizations make it the most cost-effective work organization model (e.g., Bloom et al., 2022). However, many company leaders are concerned about productivity and the possible erosion of corporate culture due to remote working arrangements and are wary of the challenges involved in hiring and onboarding remote workers (Ferreira et al., 2021).

The COVID-19 pandemic forced many businesses to move towards remote work models and accelerated the digital transformation of work — changes which partially persisted even in the absence of pandemic circumstances (Nagel, 2020; Ng et al., 2021). Before the pandemic, only 5.4% of individuals worked exclusively from home in the EU-27 (Milasi et al., 2021), nearly identical to the 5% rate among US employees (Coate, 2021). A working paper on the evolution of work from home by Barrero et al. (2023) reports that 10% of the observed US workforce is now working fully remotely. The existing literature does not comprehensively address the effects of remote work as necessitated by the COVID-19 pandemic (Waizenegger et al., 2020; Singh et al., 2022). This gap, which our inquiry aims to address, is crucial for organizations aiming to optimize remote work arrangements as a matter of company policy rather than self-selection into remote work.

In contrast to Bloom et al.’s (2022) study on the hybrid model and Bloom et al.’s (2015) study of remote work arrangements, both of which explored effects among a subset of volunteers among the employees of the studied companies, our study is based on a survey of the employees of a company that has, in response to external pressures, strategically shifted the majority of its employees to having to work exclusively from home. This working context requires additional consideration as qualitatively different from that of a volunteer-based self-selected remote or hybrid work scheme, where, for volunteers, the resources at home may be greater and the demands lesser, as understood in a work-home resources model, than of the employees more generally (Ten Brummelhuis and Bakker, 2012).

Mplus Group, a leading business process and technology outsourcing (BPTO), employs more than 13,000 individuals in 14 countries to provide contact centers, information technology, and employment services to address global customer support challenges. Before the COVID-19 pandemic, 27.8% of its employees worked from home, 4.7% worked in a hybrid manner, and 70.7% worked from the office. Since March 2020, the company has emerged as a leader in telework, with 70.7% having to work permanently from home (a 61% increase), 14.9% in a hybrid model (a 68% increase), and only 18.8% working from the office (a 276% decrease). This makes MPlus Group an appropriate case study to explore the association of working from home as the default working model with satisfaction levels, work-life balance, and working-model preferences in the BPTO sector, while additionally exploring any gender differences at a company with a woman-majority workforce.

### 1.1 Work from home and job satisfaction

The association of having to work from home (and other working models) with finding satisfaction in work is fundamental because job satisfaction is one of the key aspects of general satisfaction and quality of life (Rice et al., 1980; Montuori et al., 2022), making it important to study the possible dependence of job satisfaction on the work model. Furthermore, because employee attrition is, on average, higher in BPTO than in most industries and was a specific concern in the context of working from home amid the COVID-19 pandemic (NICE, 2022), the association of the latter with satisfaction is critical from the perspective of organizational economics. Giving greater weight to the above-discussed evidence from randomized controlled trials (Bloom et al., 2015, 2022), our first hypothesis is:

(H1) Satisfaction with work will be higher among employees working from home.

This relationship between satisfaction with work and working models may be dependent on gender. Working from home may mean providing child care during work hours, and the persistence of traditional gender roles in child-care can lead to gender differences in the ability to perform in a work-from-home set-up and affect career progression for women (Singh et al., 2022; Vaitilingam, 2022). We thus hypothesize that:

(H1a) The positive difference in satisfaction with work among employees working from home is attenuated among women.

### 1.2 Work from home and work-life balance

The greater flexibility and reduced commute-time brought about by working from home may empower employees to achieve a better work-life balance. In line with reports from previous research by Bloom et al. (2015, 2022), we hypothesize that:

(H2) Self-reported work-life balance is higher among employees working from home.

However, again due to the likely uneven distribution of home and child-care duties across genders, we hypothesize that:

(H2a) The positive difference in self-reported work-life balance among employees working from home is attenuated among women.

### 1.3 Working-model preference

Although finding satisfaction in work and work-life balance are key lenses through which to assess the implications of working from home for the employees as well as the organizations, the importance of the employee’s ability to directly express a preference for a particular working model should not be overlooked. Focusing on employees who have to work from home, we hypothesize due to the greater autonomy associated with the working model that:

(H3) Employees working from home are likely to prefer to continue working from home.

## 2 Methods

### 2.1 Procedure

In July 2022, the Mplus Group conducted a pilot study in Germany at several Invitel GmbH (a subsidiary of the Mplus Group) sites. Over 2 weeks (between the 25^th^ of July and 5^th^ of August), a distributed engagement survey was conducted based on Gallup & Willis Tower Watson methodology, comprising 40 Likert-type, categorical, and open-response questions. The pilot also assessed employee perception of survey tool confidentiality (Survey Monkey). In collaboration with 11 workers’ councils (WC) in Germany, the surveyed respondents’ highlighted cognitive load and suggested adding questions on well-being and working models (hybrid, remote, on-site) to gauge employee adjustment and motivation. Complexity reduction across countries discouraged the introduction of multidimensional constructs.

Based on feedback from the pilot study, Mplus Group streamlined the questionnaire to enhance translatability across seven additional countries and improve response rates. We prioritized respondent anonymity and the confidentiality of their perceptions. Participants were informed that the survey would take approximately 15 min to complete. The questionnaires were translated into local languages and distributed through Survey Monkey from October 10th to 23rd, 2022. The survey consisted of standardized Likert scale items and a separate analysis of open-ended questions.

### 2.2 Study design

To create a convenient sample that was still as representative as possible within the organization, 9,426 employees were invited to participate in the survey, including staff in Bosnia & Herzegovina, Croatia, Georgia, Serbia, Romania, Slovenia, and Turkey (after the pilot in Germany). Both open-ended questions and Likert-scale items were distributed in the same questionnaire, without interval differentiation. We removed respondents who were not customer experience/service and support agents (and excluded management in the same sector) to keep comparisons consistent and most pertinent to generalizations about working from home for staff within the BPTO industry.

### 2.3 Measures

Recognizing the limitations of single-item measures (Wanous et al., 1997; Nagy, 2002), we compromised to reduce questionnaire complexity and increase the sample size (Paas et al., 2003). Our quantitative analysis followed the approach of Cheung and Lucas (2014) regarding single-item measures and large samples. As a result, qualitative responses were excluded from this study.

#### 2.3.1 Job satisfaction

The Employee Engagement questionnaire was designed based on well-known methodologies (Gallup & Willis Tower Watson), and was simplified to a single item: “My work gives me a sense of personal satisfaction.” Respondents were asked to rate their perceptions from 1 (strongly disagree) to 5 (strongly agree).

#### 2.3.2 Work-life balance

The work-life balance measure was adapted from Self-Perceived Health measure developed by Eurostat (e.g., Shaaban et al., 2022), and was simplified to a single item: “I have work-life balance at my job.” Respondents were asked to rate their perceptions from 1 (strongly disagree) to 5 (strongly agree).

#### 2.3.3 Working-model preference

To assess which working model they prefer, respondents were asked to answer: “What is your understanding now, after the pandemic, of which working model (remote, hybrid, on-site) suits you best?”

### 2.4 Statistical analyses

We first estimated the simple proportion (and 95% confidence intervals, CI) of respondents stating they either “strongly agree” or “agree” with the statement “My work gives me a sense of personal satisfaction” (as opposed to not expressing a stance, disagreeing or strongly disagreeing) across the different models of work and among women and men. We introduced the distinction between required to be on-site and on-site by choice based on two questions about the working model and the reason for working on-site, in addition to the remote (which always meant working from home) and hybrid categories. We also used multilevel logistic regression models to formally test the association of the working models with personal satisfaction and adjust the estimates for the age group (18–25 as the reference group, 26–35, 36–50, 50+) and gender. To formally test whether the relation between working models and deriving satisfaction from work was dependent on gender (in addition to the simple comparison of proportions answering positively), we again used a multilevel logistic regression but included an interaction between the working model and gender.

We similarly estimated the proportion of respondents replying with either “strongly agree” or “agree” with the statement “I have work-life balance at my job” across working models and among men and women, and tested the association while adjusting for the above-mentioned covariates and in a model featuring an interaction between the working model and gender.

Finally, among the subset of respondents who were working remotely, we estimated the proportion (and 95% confidence interval) stating their preferred working model was remote (as opposed to at-the-office or hybrid); we again analyzed the association between so responding and age groups and gender using a multilevel logistic regression.

In sensitivity analyses, we took into account the original ordinal nature of the data on satisfaction and work-life balance, first with Kruskal-Wallis tests, followed by pairwise Mann–Whitney U tests for differences across work models; additionally, we analyzed the outcomes using ordinal logistic regression models, adjusting for the same covariates as in the multivariable regression models described above.

## 3 Results

Out of the 5,540 respondents (response rate of 58.8%), 4,554 worked as customer service agents and were included in the final sample. The majority worked remotely (78.77%), and fewer than 10% were required to work on-site. The largest age groups were of 18–25 years of age (45.32%) and 26–35 years of age (41.40%), with women being more prevalent in the sample, at 70.29%. Sample characteristics are summarized in Table 1.

**Table 1:** Sample characteristics.

Variable	Mean/Frequency
Work model	
From home	3,587 (78.77%)
Hybrid	252 (5.53%)
Required on-site	453 (9.95%)
On-site by choice	125 (2.74%)
NA	137 (3.01%)
Age group	
18–25	2064 (45.32%)
26–35	1885 (41.40%)
35–50	523 (11.48%)
>50	73 (1.60%)
NA	10 (0.22%)
On-site by choice	125 (2.74%)
On-site by choice	125 (0.02%)
Gender	
Women	3,201 (70.29%)
Men	1,353 (29.71%)
Country	
Bosnia and Herzegovina	305 (6.70%)
Croatia	271 (5.95%)
Georgia	23 (0.51%)
Romania	4 (0.09%)
Serbia	331 (7.27%)
Slovenia	131 (2.92%)
Turkey	3,487 (76.57%)

### 3.1 Work from home and job satisfaction (H1)

**Figure 1:**
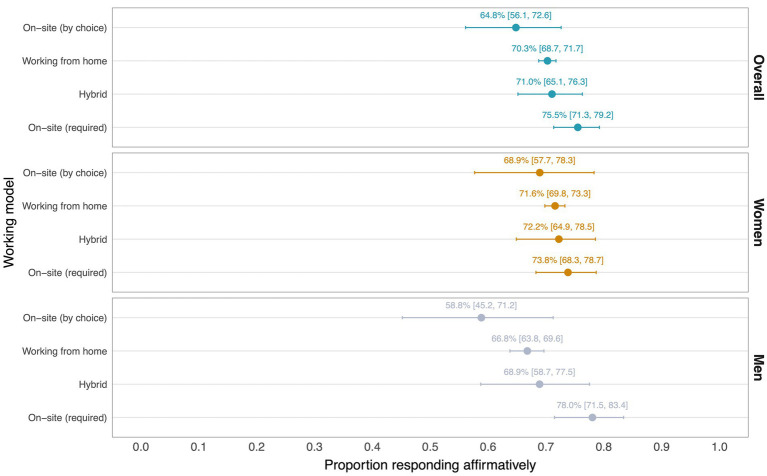
The proportion [95% confidence intervals] of respondents stating their work gives them a sense of personal satisfaction, by working model, overall and stratified by gender.

The proportion and 95% CI of respondents stating their work gives them a sense of personal satisfaction by each working model is shown in Figure 1, overall and stratified by gender.

The levels of reported satisfaction were very high and similar across all working models. Consistent with our hypothesis, respondents working from home were in fact slightly more likely to report finding satisfaction in their work (70.3, 95% CI: 68.7–71.7) than respondents choosing to work at the office (64.8, 95% CI: 56.1–72.6), although the precision of the evidence was also consistent with there being no difference (Figure 1).

**Figure 2:**
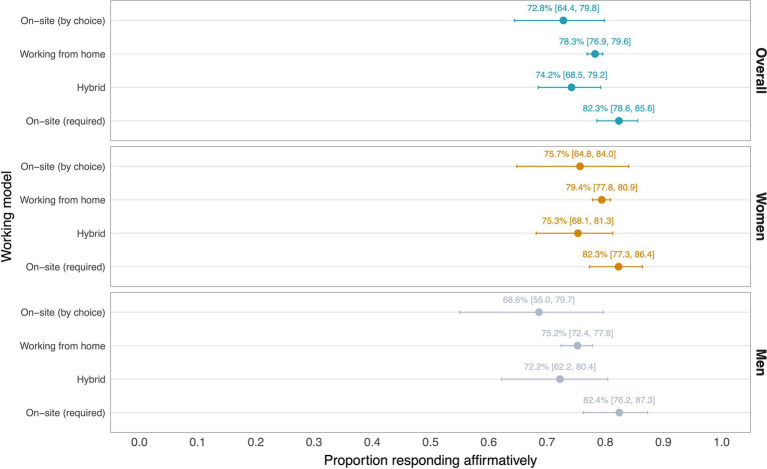
The proportion [95% confidence intervals] of respondents stating they have work-life balance, by working model, overall and stratified by gender.

The adjusted odd ratios (ORs) of finding personal satisfaction in work are reported in Supplementary Table 1 (model 1): on average, the odds were somewhat higher among respondents working from home (OR: 1.31, 95% CI: 0.89–1.91) compared with those choosing to work on-site, though the evidence was again consistent with no differences. Odds were also somewhat higher in a hybrid model (OR: 1.32, 95% CI: 0.83–2.10), and higher among those required to work on-site (OR: 1.64, 95% CI: 1.06–2.51).

Both women and men working from home reported slightly higher levels of satisfaction than those working at the office by choice, with similar uncertainty about the estimate. In the model featuring an interaction term between gender and work models and adjusting for other factors (Supplementary Table 1, model 2), men working from home were estimated to have higher odds of finding satisfaction in their work than those choosing to work at the office (OR: 1.81, 95% CI: 0.97–3.22) when adjusting for other factors. As expected, this positive association was, on average, attenuated among women working from home (OR: 0.60, 95% CI: 0.28–1.25), despite the slightly higher satisfaction among women working from home in absolute terms. Again, the evidence was also consistent with there being no difference.

Sensitivity analyses taking into account the ordinal nature of the satisfaction data (Supplementary Tables 2, 3A,B) were consistent with the above.

### 3.2 Work from home and work-life balance (H2)

The proportion and 95% CI of respondents stating they have work-life balance by each working model is shown in Figure 2, overall and stratified by gender.

The percentage reporting having work-life balance was high across working models, both for women and men. A slightly higher percentage of respondents working from home stated they had work-life balance; however, again, the evidence was also consistent with there being no difference across working models. The adjusted ORs of reporting to have work-life balance were on average higher among respondents working from home (OR: 1.29, 95% CI: 0.84–1.92) compared with those choosing to work on-site, though again these adjusted estimates were less precise (Supplementary Table 4, model 1).

In the model featuring an interaction term between gender and work models, women were estimated to have higher odds of reporting having work-life balance in their work (OR: 2.31, 95% CI: 1.02–5.28), an association that was on average attenuated among women working from home (OR: 0.46, 95% CI: 0.20–1.06) — a finding consistent with our hypothesis 2a (H2a) (Supplementary Table 4, model 2).

Sensitivity analyses taking into account the ordinal nature of the work-life balance data (Supplementary Tables 5, 6A,B) were again consistent with the analyses of the dichotomized outcome.

### 3.3 Working model preference (H3)

Among the 3,587 respondents working from home, 78% (95% CI: 0.77–0.79) stated their preferred model was to continue working from home, thus confirming our hypothesis 3 (H3). While all the age groups were more likely to prefer working from home than the youngest employees (18–25), there were no substantive differences between women and men. The estimates are presented in Supplementary Table 3. The country-specific varying intercepts from all the multilevel logistic regression models are reported in Supplementary Figures 1–5.

## 4 Discussion

### 4.1 Results in the context of previous work

This study explored the association of having to work from home with self-reported satisfaction, work-life balance, and working model preferences in a diverse sample of 4,554 employees of the MPlus Group, a leading business process and technology outsourcing company that switched in large part to remote work during the COVID-19 pandemic and did not revert to its previous distribution of work arrangements. Satisfaction and work-life balance levels were overall very high and respondents were very likely to prefer to continue working from home. These results were similar across all working models at the company, with minimal differences between women and men in the sample.

More specifically, working from home was associated with somewhat higher levels of personal satisfaction and work-life balance compared to choosing to work at the office. While the uncertainty of these estimates prevents concluding that there are genuine substantial generalizable differences, we can confidently state that working from home was not associated with substantially lower employee satisfaction or work-life balance. While women appeared to, on average, benefit less from working from home in terms of personal satisfaction and work-life balance, these estimates were uncertain, and the evidence for this difference thus not strong. Finally and critically, respondents who had to work from home were very likely to state they prefer to continue working from home.

Much of the recent research on work from home has focused on its productivity implications. In addition to informing this perspective indirectly, our study helps answer the more straightforward questions about the implications of having to work from home on the well-being of the working population in the BPTO and similar industries. Aksoy et al. (2023) reported that workers saved 72 min daily on commuting, allocating 40% of their time savings to work, 34% to leisure, and 11% to caregiving activities, which may help understand the positive associations with working from home observed in this study.

The results of this study are also not surprising in the context of the two randomized controlled trials of volunteers assigned to hybrid and remote work, respectively by Bloom et al.’s (2022) and Bloom et al. (2015); however, the positive differences in job satisfaction and work-life balance observed in the present study were considerably smaller and thus statistically uncertain. This difference could be explained in part by the distinctive feature of this study — the fact that the sub-sample of the employees working from home in the present study did not self-select to participate in an experiment, but continued working from home as a continuation of a policy introduced and deemed successful by the company’s management during the COVID-19 pandemic. On the other hand, the results stand in contrast with those of other observational studies, such as those by Makridis and Schloetzer (2022) and Niebuhr et al. (2022), which found negative associations with satisfaction.

With respect to the estimated negative interaction of gender with working from home, the uncertainty of the estimates precludes confident conclusions. However, considering absolute values, the clearly high level of satisfaction, work-life balance, and preference to continue working from home observed among women in this study are perhaps unexpected given previous research (Singh et al., 2022) and are encouraging in terms of equity as well as enhanced productivity considerations (Yang et al., 2022).

### 4.2 Limitations and future research

This study has several important limitations. Despite the large and culturally diverse sample, appropriate generalizations based on this study may be limited to similar industries. Further, the single-item measures may not reflect the complexity of the studied phenomena, but were chosen to simplify the conduct of the survey and interpretation. One of the distinguishing features of the study, i.e., the fact that the respondents were not self-selected as volunteers but were rather regularly working from home as a consequence of the company’s policy (Yu and Wu, 2021), also means that working from home was not randomized — the results, therefore, fall short of addressing questions about the causal effects of having to work from home. A longitudinal exploration of each of the questions addressed here would also have been informative, especially if it included the period before the shift of many respondents to working from home. Exploring the relation between remote work and employees’ motivation more generally, and as reflected in burnout and turnover, may be another valuable research avenue, possibly as understood through Self-determination theory (Deci et al., 2017; Tudu and Singh, 2022).

## 5 Conclusion

This brief research report examined the association of having to work from home with job satisfaction, work-life balance, and working model preference within the BPTO industry. Both women and men in all working models, including fully remote work from home, reported high levels of job satisfaction and work-life balance. Respondents working from home were also very likely to prefer continuing to do so. Overall, this study contributes to the ongoing discussions on remote work and provides insights to organizations and policymakers navigating the changing landscape of post-pandemic work dynamics. Further research is needed to explore working from home arrangements in other industries, as well as to study the phenomenon longitudinally.

## Data availability statement

The datasets presented in this article are not readily available because sharing them may make certain participants easily identifiable. Requests to access the datasets should be directed to tin.oreskovic@balliol.ox.ac.uk.

## Ethics statement

Ethical approval was not required for the studies involving humans because it was based entirely on a survey conducted by the HR department of a company. The studies were conducted in accordance with the local legislation and institutional requirements. The participants provided their written informed consent to participate in this study.

## Author contributions

TO: Conceptualization, Formal Analysis, Methodology, Software, Visualization, Writing – original draft, Writing – review & editing. MM: Conceptualization, Formal Analysis, Methodology, Software, Writing – review & editing. BK: Conceptualization, Data curation, Project administration, Writing – review & editing. DH: Conceptualization, Data curation, Project administration, Writing – review & editing. TG: Conceptualization, Data curation, Project administration, Writing – review & editing. AS: Conceptualization, Methodology, Writing – original draft, Writing – review & editing. C-IK: Conceptualization, Writing – review & editing, Supervision. SO: Conceptualization, Methodology, Project administration, Supervision, Writing – original draft, Writing – review & editing.
