# Supplementary material for: Associations of working from home with job satisfaction, work-life balance, and working-model preferences
Source: Front Psychol. 2023 Nov 22;14:1258750. doi: 10.3389/fpsyg.2023.1258750 (PMC10702569; doi:10.3389/fpsyg.2023.1258750)
Supplement: Supplementary file 1 [file Table_1.DOCX]

Supplementary Material

# Supplementary Figures and Tables

Supplementary Table 1. Multilevel logistic regression: getting personal satisfaction from work

| **Variable** | **Satisfaction model 1**  **(OR [95% CI])** | **Satisfaction model 2**  **(OR [95% CI])** |
| --- | --- | --- |
| **Work model** (compared to on-site by choice):  **From home** | 1.31 (0.89-1.91) | 1.81 (0.97-3.22) |
| **Hybrid** | 1.32 (0.83-2.10) | 2.42 (1.10-5.48) |
| **Required on-site** | 1.64 (1.06-2.51) | 2.13 (1.05-4.26) |
| **Age group** (compared to 18-25):  **26-35** | 0.86 (0.74-0.99) | 0.85 (0.74-0.98) |
| **36-50** | 0.97 (0.77-1.21) | 0.96 (0.77-1.20) |
| **50+** | 1.41 (0.82-2.56) | 1.38 (0.80-2.50) |
| **Gender** (compared to men):  **Women** | 1.00 (0.86-1.15) | 1.69 (0.79-4.84) |
| **Women*Home** | - | 0.60 (0.28-1.25) |
| **Women*Hybrid** | - | 0.41 (0.15-1.08) |
| **Women*Required on site** | - | 0.66 (0.27-1.51) |
| N=4409 |  |  |

OR: odds ratio; 95% CI: 95% confidence interval

**Supplementary Table 2: False-discovery-rate P-values from pairwise Mann-Whitney U tests for differences in satisfaction (ordinal) across work models**

| **Work model** | **On-site by choice** | **From home** | **Hybrid** |
| --- | --- | --- | --- |
| **From home** | 0.69 | - | - |
| **Hybrid** | 0.69 | 0.73 | - |
| **Required on-site** | 0.08 | 0.002 | 0.10 |

Kruskal Wallis chi-squared = 13.715, df = 3, p-value = 0.003

Supplementary Table 3A: Ordinal logistic regression: getting personal satisfaction

from work

| **Variable** | **(OR [95% CI])** |
| --- | --- |
| **Work model** (compared to on-site by choice):  **From home** | 1.13 (0.81-1.57) |
| **Hybrid** | 1.13 (0.76-1.68) |
| **Required on-site** | 1.54 (1.07-2.23) |
| **Age group** (compared to 18-25):  **26-35** | 0.81 (0.72-0.91) |
| **36-50** | 0.81 (0.68-0.97) |
| **50+** | 1.25 (0.79-1.96) |
| **Gender** (compared to men):  **Women** | 1.05 (0.94-1.19) |

OR: odds ratio; 95% CI: 95% confidence interval

Supplementary Table 3B: Thresholds from the ordinal satisfaction model

| **Threshold** | **Value** |
| --- | --- |
| **Strongly disagree – Disagree** | -2.75 |
| **Disagree – Neither agree nor disagree** | -1.82 |
| **Neither agree nor disagree – Agree** | -0.81 |
| **Agree – Strongly agree** | 0.80 |

Supplementary Table 4: Multilevel logistic regression: work-life balance

| **Variable** | **Work-life balance model 1**  **(OR [95% CI])** | **Work-life balance model 2**  **(OR [95% CI])** |
| --- | --- | --- |
| **Work model** (compared to on-site by choice):  **From home** | 1.29 (0.84-1.92) | 2.06 (1.07-3.84) |
| **Hybrid** | 1.04 (0.63-1.69) | 1.79 (0.79-4.08) |
| **Required on-site** | 1.61 (1.00-2.56) | 2.59 (1.23-5.43) |
| **Age group** (compared to 18-25):  **26-35** | 1.08 (0.92-1.26) | 1.07 (0.92-1.26) |
| **36-50** | 0.96 (0.75-1.22) | 0.95 (0.75-1.21) |
| **50+** | 1.21 (0.69-2.20) | 1.18 (0.67-2.15) |
| **Gender** (compared to men):  **Woman** | 1.09 (0.93-1.26) | 2.31 (1.02-5.28) |
| **Women*Home** | - | 0.46 (0.20-1.06) |
| **Women*Hybrid** | - | 0.41 (0.14-1.16) |
| **Women*Required on-site** | - | 0.45 (0.17-1.19) |
| N=4409 |  |  |

OR: odds ratio; 95% CI: 95% confidence interval

**Supplementary Table 5: False-discovery-rate P-values from pairwise Mann-Whitney U tests for differences in having work-life balance (ordinal) across work models**

Kruskal Wallis chi-squared = 13.715, df = 3, p-value = 0.003

| **Work model** | **On-site by choice** | **From home** | **Hybrid** |
| --- | --- | --- | --- |
| **From home** | 0.74 | - | - |
| **Hybrid** | 0.80 | 0.74 | - |
| **Required on-site** | 0.20 | 0.16 | 0.20 |

Kruskal-Wallis chi-squared = 5.9491, df = 3, p-value = 0.1141

Supplementary Table 6A: Ordinal logistic regression: having work-life balance

| **Variable** | **(OR [95% CI])** |
| --- | --- |
| **Work model** (compared to on-site by choice):  **From home** | 1.15 (0.82-1.62) |
| **Hybrid** | 1.07 (0.71-1.61) |
| **Required on-site** | 1.39 (0.96-2.02) |
| **Age group** (compared to 18-25):  **26-35** | 0.94 (0.83-1.06) |
| **36-50** | 0.77 (0.64-0.92) |
| **50+** | 1.15 (0.73-1.82) |
| **Gender** (compared to men):  **Women** | 1.03 (0.91-1.16) |

OR: odds ratio; 95% CI: 95% confidence interval

Supplementary Table 6B: Thresholds from the ordinal work-life balance model

| **Threshold** | **Value** |
| --- | --- |
| **Strongly disagree – Disagree** | -2.85 |
| **Disagree – Neither agree nor disagree** | -2.10 |
| **Neither agree nor disagree – Agree** | -1.17 |
| **Agree – Strongly agree** | 0.64 |

## Supplementary Figures

Supplementary Figure 1: Varying intercepts from Satisfaction model 1

Supplementary Figure 2: Varying intercepts from Satisfaction model 2

Supplementary Figure 3: Varying intercepts from Work-life balance model 1

Supplementary Figure 4: Varying intercepts from Work-life balance model 2

Supplementary Figure 5: Varying intercepts from preferring to continue working from home model

**
